# Supplementary material for: The effect of a cognitive dual-task on the control of wheelchair propulsion
Source: PLoS One. 2025 Feb 13;20(2):e0317504. doi: 10.1371/journal.pone.0317504 (PMC11824953; doi:10.1371/journal.pone.0317504)
Supplement: S1 Table — Note: significant differences between manual wheelchair users and novices are marked by ‘*’. Due to the lack of a normal distribution, the Mann-Whitney U-test and Wilcoxon test were used to evaluate these parameters. (PDF) [file pone.0317504.s001.pdf]

S1 Table. Comparison of the mean spatiotemporal propulsion parameters variability during single- and dual-task driving a wheelchair.

	**Single-Task**		**Dual-Task**		**ST vs DT** (p-value)	
	manual wheelchair users	*novices*	*manual wheelchair users*	*novices*	*manual wheelchair users*	*novices*
**cycle time variability [s]**	0,04 ± 0,02	0,04 ± 0,02	0,05 ± 0,03	0,08 ± 0,14	0,577	0,551
**push time variability [s]**	0,03 ± 0,01	0,03 ± 0,02	0,03 ± 0,02	0,07 ± 0,15	0,049	0,331
**recovery time variability [s]**	0,03 ± 0,01	0,04 ± 0,02	0,04 ± 0,02	0,04 ± 0,04	0,071	0,730
**push angle variability [deg]**	3,8 ± 2,0	3,4 ± 0,7	4,3 ± 2,6	3,0 ± 1,0*	0,162	0,084
**distance rim variability [cm]**	1,5 ± 0,8	1,1 ± 0,6	1,4 ± 0,8	1,1 ± 0,7	0,357	0,875
**distance axis variability [cm]**	1,1 ± 0,9	0,3 ± 0,2*	0,7 ± 0,7	0,3 ± 0,1	0,036	0,683

Note: significant differences between manual wheelchair users and novices are marked by ''. Due to the lack of a normal distribution, the Mann-Whitney U-test and Wilcoxon test were used to evaluate these parameters.*
